# Feeding Intolerance and Citrulline Generation Test in the Critically Ill: A Prospective Study

**DOI:** 10.3390/nu18172759

**Published:** 2026-08-24

**Authors:** Chloé Hild, Thibault Vieille, Marc Puyraveau, Hadrien Winiszewski, Karena Moretto Riedweg, Gaël Piton

**Affiliations:** 1 Université Marie et Louis Pasteur, CHU Besançon, SINERGIES, 25000 Besançon, France; cgaude@chu-besancon.fr (C.H.); hwiniszewski@chu-besancon.fr (H.W.); 2 Medical Intensive Care Unit, Besançon University Hospital, 25030 Besançon, France; tvieille@chu-besancon.fr; 3 Clinical Methodology Unit, Besançon University Hospital, 25030 Besançon, France; mpuyraveau@chu-besancon.fr; 4 Biochemistry Unit, Besançon University Hospital, 25030 Besançon, France; kriedweg@chu-besancon.fr

**Keywords:** feeding intolerance, critically ill, citrulline generation test

## Abstract

**Background/Objectives**: Early feeding intolerance (FI) affects 20–50% of critically ill patients receiving enteral nutrition, yet no validated predictive tool exists and diagnosis remains retrospective. Plasma citrulline, mainly synthesized by enterocytes, is considered as the “factor V of the intestine”. The citrulline generation test (CGT) evaluates small bowel mucosal function by measuring the increase in plasma citrulline after glutamine administration. We hypothesized that patients with a lower CGT would be more prone to FI. We aimed to compare CGT results according to the presence or absence of FI and to identify factors associated with CGT. **Methods**: This prospective study was conducted in the medical intensive care unit (ICU) of Besancon Hospital. Plasma citrulline and glutamine concentrations were measured at admission and after an alanine–glutamine dipeptide bolus. CGT was defined as the slope between basal and 90 min peak plasma citrulline concentrations. Signs of FI were collected during follow-up. **Results**: Among the sixty-six included patients, nine (14%) developed FI by day three, only characterized by vomiting and gastric residual volumes above 500 mL; abdominal pain or diarrhea were not observed. CGT values did not differ between patients with and without FI. CGT correlated positively with baseline citrulline and peak glutamine concentrations. **Conclusions**: FI was not associated with CGT, possibly because FI was predominantly gastric rather than intestinal. Low CGT values were associated with low plasma citrulline concentrations, suggesting reduced functional enterocyte mass. The positive correlation between CGT and peak plasma glutamine suggests that CGT not only reflects enterocyte function but also glutamine bioavailability.

## 1. Introduction

Nutrition is a piece of the puzzle of intensive care unit (ICU) care and the recent literature emphasizes the importance of timing, route, and amount of nutrition among critically ill patients [1]. However, 20 to 50% of critically ill patients receiving enteral nutrition (EN) will experience early feeding intolerance (FI), defined as the inability to receive the correct amount of EN due to vomiting, regurgitation, abdominal pain, or diarrhea, and therefore requiring EN interruption [2]. There is no validated tool for the prediction of FI among critically ill patients, and the diagnosis is always performed a posteriori, while under EN.

Plasma citrulline is an amino acid mainly synthesized by enterocytes and is considered as a biomarker of functional enterocyte mass in stable conditions [3,4,5]. Indeed, among patients with chronic small bowel disease, plasma citrulline concentration correlates with remnant small bowel length after small bowel resection, and with villi length among patients presenting with villous atrophy-associated small bowel diseases. In other words, plasma citrulline is considered by gastroenterologists as the “factor V of the intestine”. However, the relevance of plasma citrulline concentration among critically ill patients remains a subject of debate. The link between low plasma citrulline concentration and increased mortality is not clear among critically ill patients, whereas it is clearly established that sepsis and systemic inflammation is related to low plasma citrulline concentration [6,7,8]. The complexity of the interpretation of a single measurement of plasma citrulline concentration in the context of critical disease has led Peters and al. to propose a new diagnostic test called the “citrulline generation test” (CGT) [9]. Briefly, the CGT explores the ability of the small bowel mucosa to produce citrulline after the administration of its main precursor, i.e., glutamine, given orally or intravenously, and evaluated by the slope of citrulline elevation over time. The CGT could have the advantage of combining a static test, regarding the basal citrulline concentration before the glutamine bolus; and a dynamic test, regarding the slope of citrulline elevation over time after the glutamine bolus. These two parameters could help to evaluate the functional enterocyte mass among critically ill patients. However, the value of the CGT for the evaluation of small bowel function and the ability of critically ill patients to receive EN is unknown. We hypothesized that patients with a lower CGT result would have a higher rate of FI compared to patients with a normal CGT result. The primary objective was to compare the CGT results among patients with and without FI. The CGT was conducted before EN initiation. FI was assessed during the first three days of EN. The secondary objective was to identify the factors associated with the CGT among critically ill patients.

## 2. Materials and Methods

**Aim**: The primary objective was to compare the CGT results among patients with and without FI. The CGT was conducted before EN initiation. FI was assessed during the first three days of EN. Secondary objectives were to explore the factors associated with the CGT among critically ill patients.

**Setting**: Intensive Care Unit in a University Hospital.

**Inclusion criteria**: Critically ill adult patients requiring ICU admission, with indication of EN, and a predicted duration of ICU stay ≥48 h.

**Exclusion criteria**: Chronic small bowel disease (inflammatory bowel disease, celiac disease, or history of small bowel resection); chronic renal failure defined by a clearance of creatinine <50 mL/min; pregnancy; contraindication to EN (in particular, admission after bowel surgery or for acute mesenteric ischemia).

**Variables studied**:

*Demographic variables*: age, sex, body mass index.

*Clinical variables*: heart rate, mean arterial pressure, SpO_2_.

*Biological variables*: plasma creatinine concentration, plasma creatinine clearance, C-reactive protein (CRP), and plasma lactate concentrations.

*Variables of treatment at ICU admission*: catecholamine use, norepinephrine dose, mechanical ventilation, inspired fraction of oxygen, continuous veno-venous hemodiafiltration.

*Variables of prognosis*: SOFA score [10], 28-day mortality rate.

*Enterocyte biomarkers*: Plasma glutamine and citrulline concentrations were assessed by ultra performance liquid chromatography coupled with tandem mass spectrometry at basal time and after a simplified CGT. At ICU admission, a first arterial sampling was performed on a heparin tube (4 mL) for the plasma citrulline concentration (citrullinemia 1). Ninety minutes after the intravenous bolus of glutamine, a second arterial sampling was performed (citrullinemia 2). After centrifugation (10 min at 2250× *g* and 4 °C), an aliquoting was achieved (at least 200 μL of plasma per cryotube) in two cryotubes. Then, cryotubes were vortexed and stored at −80 °C until analysis. Amino acids analysis required deproteinization, sample preparation by hydrolysis and derivatization (AccQ Tag^®^, Waters Corporation, Milford, MA, USA), separation using reverse-phase ultra performance liquid chromatography, and detection with a tandem mass spectrometer. This was achieved in a reference laboratory within the hospital.

*Simplified Citrulline Generation Test*: The CGT consisted in a simplified CGT with two-point slope, as previously described by Peters [9,11]. Two hundred milliliters of saline solution was added to 100 mL of DIPEPTIVEN^®^ (Fresenius Kabi France, Sèvres, France) containing 20 g of the dipeptide alanine–glutamine. It was administered intravenously over a period of 30 min through a central or peripheral venous catheter. Citrullinemia at basal time (T0) and 90 min after the end of the glutamine bolus (T90) were measured on an arterial catheter. Peters et al. have shown that an intravenous administration of glutamine and an arterial plasma citrulline sampling are more appropriate and reliable in critically ill patients, and that a two-point test (T0 and T90) is applicable [9,11]. The slope between the basal citrullinemia and the peak was calculated (citrullinemia T90-citrullinemia T0)/90 and expressed in µmol/L/min. Clinical signs of hypersensitivity secondary to the DIPEPTIVEN^®^ infusion were collected.

*Protocol of enteral nutrition (EN)*: EN was started within 24 h (if possible) after the second sampling of citrullinemia. Two products could be used: a high-protein, high-energy formula [NUTRISON PROTEINE PLUS ENERGY^®^ (1.5 kcal/mL and 7.5 g protein/100 mL) (Nutricia Nutrition Clinique, Rueil-Malmaison, France)] or a peptide-based, semi-elemental, high-protein formula [PEPTAMEN INTENSE^®^ (1 kcal/mL and 9.3 g protein/100 mL) (Nestle Health Science France, Issy-les-Moulineaux, France)]. It was administered on a nasogastric tube, with a continuous flow, according to a standardized protocol as recommended by expert societies, i.e., 16 kcal/kg/d and 1 g/kg/d of protein during the first day in ICU, 24 kcal/kg/d and 1.5 g/kg/d of protein between the second and the seventh day, 36 kcal/kg/d and 2 g/kg/d of protein after the seventh day.

*Feeding Intolerance (FI)*: During the patient’s follow-up, we collected FI signs at day 3 and day 7. FI was defined by at least one of the following signs or symptoms: vomiting or regurgitation during EN, gastric residual volume more than 500 mL once, abdominal pain or profuse diarrhea requiring EN cessation. Digestive complications related to EN were collected: acute mesenteric ischemia, acute colonic pseudo-obstruction, aspiration pneumonia. Acute Gastro Intestinal Score was recalculated a posteriori [12].

**Information and consent**: Written informed consent was obtained from patients or from their relatives when patients were unable to consent.

**Statistical analysis**: Qualitative variables were expressed as number (percentage) and compared with the Fischer’s exact test. Quantitative variables were expressed as median [interquartile range] and compared with the Mann–Whitney test or with the Kruskal–Wallis test. Correlation between two quantitative variables was performed using the Spearman correlation test. A *p* value of <0.05 was considered statistically significant. All statistical analyses were performed using IBM SPSS Statistics 27.0.

**Registration**: This study was registered on ClinicalTrials.Gov as the number NCT03967795 (registration date: 28 November 2019).

## 3. Results

### 3.1. Description of the Population

Seventy-three patients were initially included: three were excluded due to consent removal; two patients were subsequently excluded because of initially unknown guardianship; one patient was excluded because biological sampling for plasma amino acid determination was not performed; one patient was excluded because he was discharged rapidly from the ICU and did not receive enteral nutrition. Finally, 66 patients were included in the analysis. Table 1 summarizes the characteristics of the critically ill patients at admission and of the CGT.

### 3.2. Characteristics of FI

The description of FI appears in Table 2.

FI was observed in 14% of patients during the first three days and 26% during the first week. The main symptoms of FI were vomiting and gastric residual volumes (GRVs) greater than 500 mL. No case of abdominal pain or diarrhea while under EN was observed.

### 3.3. Comparison of CGT According to FI

**Figure 1:**
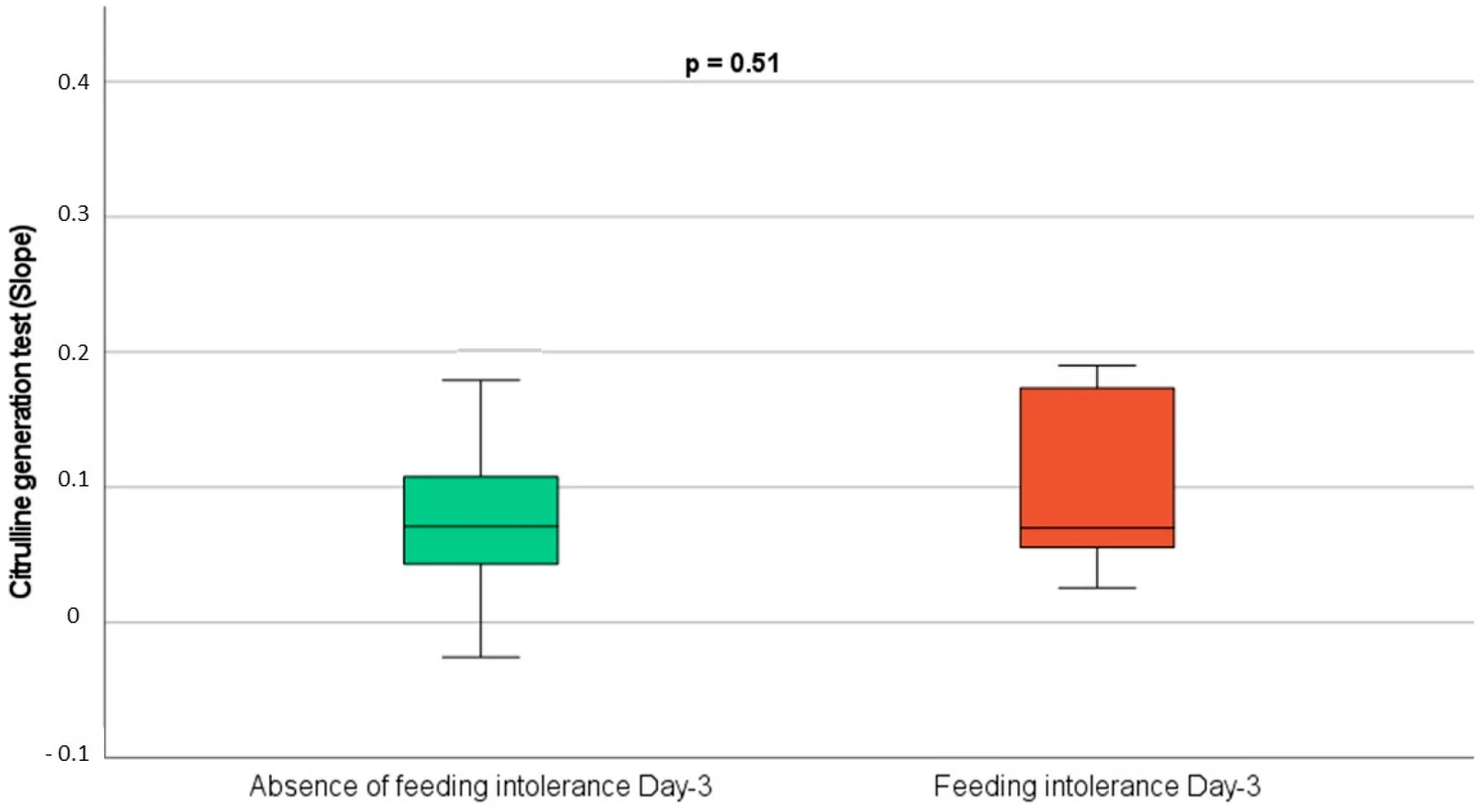
Univariate analysis of the citrulline generation test according to the presence or absence of feeding intolerance at day 3 among 66 critically ill patients.

There was no difference in CGT between patients with and without FI, as shown in Figure 1.

No difference was observed for CGT and for basal plasma citrulline concentration among the four different contexts of ICU admission (Table 3).

### 3.4. Univariate Analysis of Factors Associated with Different Classes of CGT

This analysis is shown in Table 4. CGT thresholds have been suggested in previous studies. Significant differences were observed between the peak plasma glutamine concentration and the four CGT classes, as well as between basal and peak plasma citrulline concentrations and the four CGT classes.

### 3.5. Univariate Correlation Between CGT and Biological Variables of Interest

**Figure 2:**
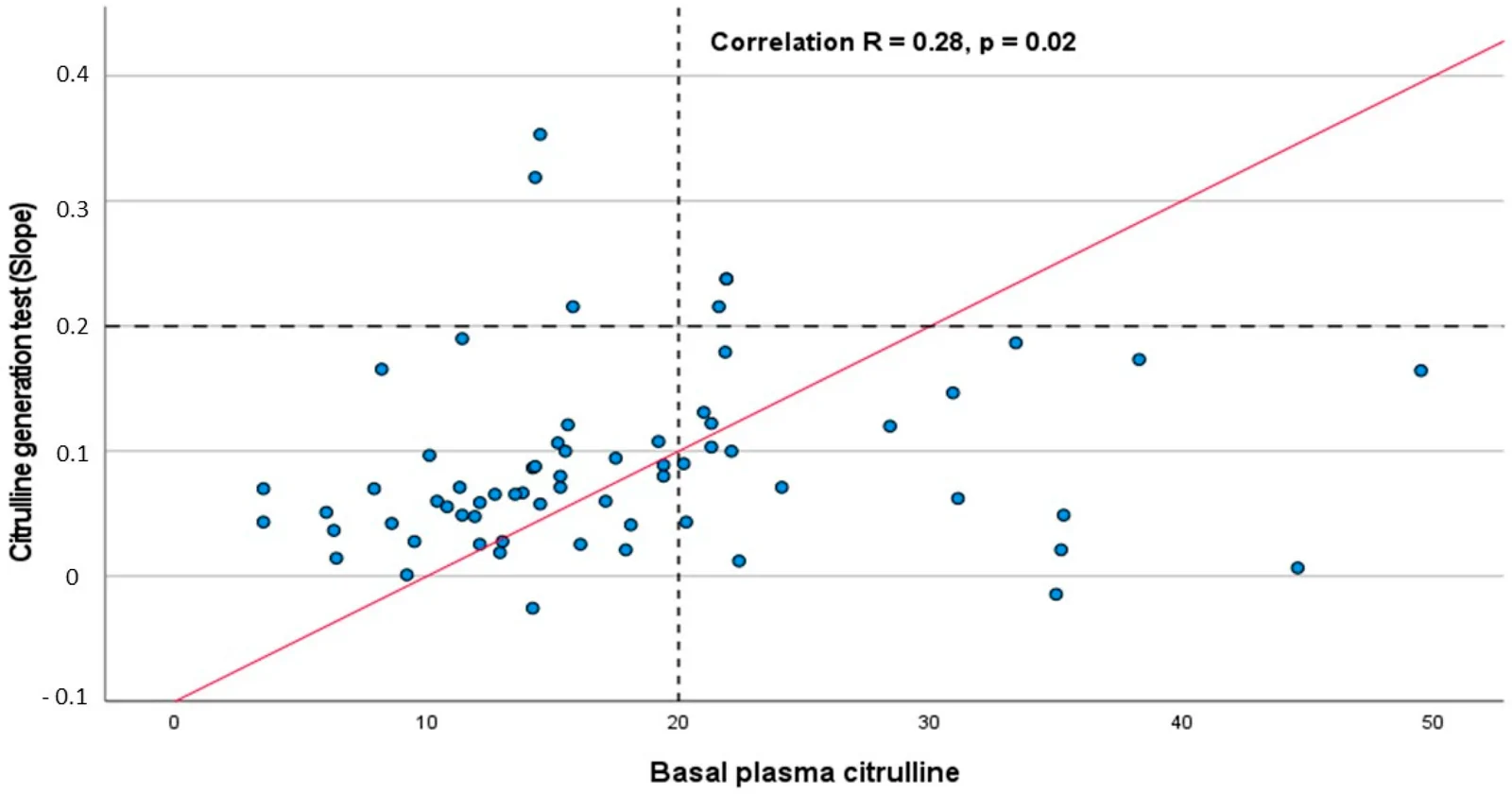
Correlation between basal plasma citrulline concentration and the citrulline generation test among 66 critically ill patients. The dash lines correspond to the thresholds of normal plasma citrulline concentration (≥20 µmol/L) and normal CGT values (≥0.20 µmol/L/min).

**Figure 3:**
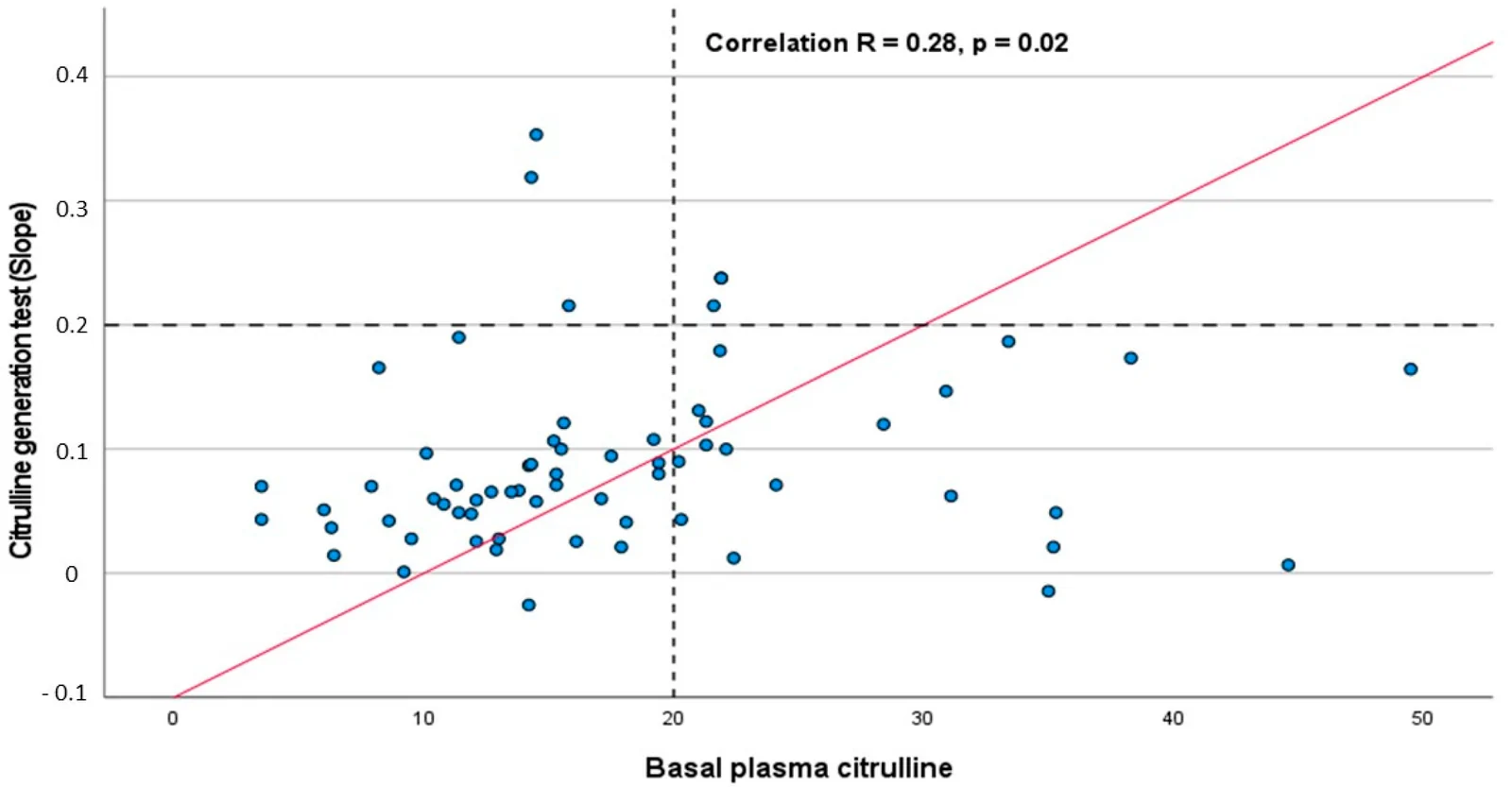
Correlation between the peak of plasma glutamine concentration at 90 min and the citrulline generation test among 66 critically ill patients. The dash line corresponds to the threshold of normal CGT values (≥0.20 µmol/L/min).

This analysis is shown in Table 5, Figure 2 and Figure 3. CGT showed a significant positive correlation with basal plasma citrulline concentration and peak plasma glutamine concentration.

### 3.6. Univariate Analysis of Factors Associated with Feeding Intolerance

This analysis is shown in Table 6.

## 4. Discussion

The four main results of this study are the followings: we observed no correlation between FI and the CGT, but the clinical characteristics of FI related only to gastric signs of intolerance; the CGT result was very low among critically ill patients; the CGT positively correlated to basal citrulline; the CGT positively correlated to the peak of glutamine.

The first result of this study is that, contrary to our primary hypothesis, we observed no correlation between the CGT performed before initiation of EN and the presence of FI the first three days of EN in the ICU. FI occurred in 14% and 26% of the patients at day 3 and day 7 of EN, respectively. Interestingly, in this study, FI only accounted for gastric signs of intolerance: vomiting and/or increased gastric residual volume. On the contrary, there was no sign of FI that could involve a lower part of the digestive tract, such as diarrhea or acute mesenteric ischemia. One could hypothesize that the CGT does not explore the gastric function, but the small bowel function. Pathophysiologically, there is likely a rationale for a distinction between the different levels of FI, with upper/gastric dysfunction, medium/small bowel dysfunction, and lower/colon dysfunction.

The second result of this study is that the CGT results were extremely low, with a median slope of 0.07 µmol/L/min, in a population of severe critically ill patients. We used a simplified CGT with a two-point measurement of plasma citrulline, as proposed by Peters [11]. Peters et al. described a CGT value of 0.22 µmol/L/min among healthy volunteers [9]. Therefore, a normal CGT value is likely to be ≥0.20 µmol/L/min. The only other study having described the CGT in the ICU was that of Peters, performed among 16 critically ill patients [11]. In that study, the median slope of citrulline increase was approximately of 0.20 µmol/L/min, which was not different from a previous description of the CGT among healthy adults. To the best of our knowledge, we report the higher number of critically ill patients in whom the CGT was performed. In addition, this is the first study comparing the CGT results among critically ill patients with and without FI. The CGT results described in the present study were approximately three times lower than those described among critically ill patients by Peters. However, the patients included in the present study were significantly more severe than in Peters’ study, with higher SOFA score and mortality rate. In another study by Peters, patients presenting with a severe celiac disease had very low CGT value, lower than 0.10 µmol/L/min, which was similar to our study [9]. These results reinforce the hypothesis that there is a reduced functional enterocyte mass among critically ill patients and that the global severity matters. Indeed, many factors may account for a reduced enterocyte mass among critically ill patients, in particular shock and non-occlusive mesenteric ischemia leading to enterocyte ischemia, but also systemic inflammation leading to enterocyte apoptosis [8,13].

The third result of this study is that the CGT correlated with basal citrulline: the lower the basal citrulline, the lower the slope of citrulline increase after a bolus of glutamine. Physiologically, there is a rationale to assume that patients having a reduced functional enterocyte mass have a low basal plasma citrulline concentration, and that their ability to increase citrulline after a bolus of glutamine is also reduced, leading to a reduced CGT value. This suggests that both biomarkers, basal plasma citrulline concentration and the slope of the CGT, give concordant information on a possible reduction in the functional enterocyte mass.

The fourth result of this study is that the CGT correlated with the peak of glutamine measured 90 min after the intravenous bolus, but not with the basal plasma glutamine concentration. In our opinion, this result is very interesting because it suggests that the CGT does not only explore the enterocyte level and the ability of the enterocyte to produce citrulline. This result suggests that the CGT could also be dependent on the pre-enterocyte level, particularly the glutamine bioavailability, as a source of precursor for citrulline synthesis.

This study had several limitations. First, as an exploratory study, no sample size calculation was performed. With only 73 patients (and missing data for several variables), this study sample may be insufficient to detect small but clinically meaningful differences. Second, we did not use the AGI classification in the study protocol [12]. However, recalculation of the AGI score showed that all of the patients presenting with FI during the first three days of EN had an AGI score equal to 2. There was no case of AGI 4, corresponding to digestive emergency. We cannot exclude that the CGT could be lower among patients with a higher AGI score (3 or 4) compared with lower AGI scores (≤2).

## 5. Conclusions

In conclusion, we observed no correlation between FI and the CGT, but the patients presented only with gastric signs of FI, whereas the CGT may explore the small bowel function. The CGT value was very low among critically ill patients, three times lower than that described in a healthy population, and similar to that described among patients with severe celiac disease, suggesting a reduced functional enterocyte mass among them. A lower basal citrulline concentration correlated to a lower CGT value, suggesting that both basal citrulline and CGT may give concordant information on the level of the functional enterocyte mass. A higher peak of glutamine correlated to a higher CGT value, raising the hypothesis that the CGT could explore more than the functional enterocyte mass and could be dependent on the glutamine bioavailability for the enterocytes.

## Figures and Tables

**Table 1:** Characteristics of the critically ill patients at inclusion and characteristics of the citrulline generation test.

Variable	*n* = 66
*Clinical variables*	
Age (years)	67 [58–75]
Male sex	40 (61)
Chronic heart failure	4 (6)
Chronic respiratory failure	7 (11)
Cirrhosis	2 (3)
Body mass index (kg/m^2^)	28 [25–32]
Heart rate (bpm)	86 [73–106]
Mean arterial pressure (mmHg)	84 [73–96]
SpO_2_ (%)	96 [94–98]
Digestive bleeding	0 (0)
Diarrhea	0 (0)
*Indication for ICU admission*	
Cardiac arrest	13 (20)
Neurological failure	12 (18)
Shock	14 (21)
Respiratory failure	27 (41)
*Biological variables*	
Creatinine (µmol/L)	101 [67–139]
Creatinine clearance (mL/min)	60 [43–97]
C-reactive protein (mg/L)	74 [17–186]
Lactate (mmol/L)	1.7 [1.3–3.4]
*Variables of treatment*	
Norepinephrine use	23 (35)
Norepinephrine dose (µg/kg/min)	0 [0–0.22]
Mechanical ventilation	47 (71)
FiO_2_ (%)	75 [50–100]
CVVHDF	7 (11)
Enteral nutrition	
Delay of initiation of EN (hours)	41 [22–57]
Caloric intake day 1 (kcal/kg/d)	8 [6–10]
Caloric intake day 2 (kcal/kg/d)	15 [6–21]
Caloric intake day 3 (kcal/kg/d)	15 [8–22]
*Variables of prognosis*	
SOFA score	8 [6–11]
28-day mortality	25/64 (39)
*Variables associated with the citrulline generation test*	
Hypersensitivity to DIPEPTIVEN©	0 (0)
Glutamine (µmol/L)	
Baseline	481 [379–633]
Peak	877 [600–1264]
Citrulline (µmol/L)	
Baseline	15.4 [11.9–21.6]
Peak	23.3 [16.2–33.7]
Slope (simplified 2-points CGT)	0.07 [0.04–0.12]

Numbers are N (%) and median [interquartile range]. CVVHDF, continuous veno-venous hemodiafiltration; SOFA, sequential organ failure assessment.

**Table 2:** Description of feeding intolerance during the first week in the ICU.

Variables	Day 1	Day 2	Day 3	Day 4	Day 5	Day 6	Day 7
Presence of FI	1/66 (2)	5/66 (8)	6/63 (10)	3/57 (5)	4/47 (9)	2/44 (5)	2/38 (5)
Vomiting	0	1	3	2	2	1	2
Regurgitation	0	0	0	0	0	0	0
Single GRV > 500 mL	1	4	4	1	3	2	0
Abdominal pain	0	0	0	0	0	0	0
Profuse diarrhea with EN interruption	0	0	0	0	0	0	0
FI the first three days	9/66 (14)						
FI the first week	17/66 (26)						
AGI score the first three days							
<2	57 (86)						
2	9 (14)						
3	0 (0)						
4	0 (0)						
Digestive complication	0 (0)						

Numbers are N (%). AGI, acute gastro-intestinal; EN, enteral nutrition; FI, feeding intolerance; GRV, gastric residual volume; ICU, intensive care unit.

**Table 3:** Plasma citrulline concentration and citrulline generation test according to the indication of ICU admission.

Variables	Cardiac Arrest	Neurological Failure	Shock	Respiratory Failure	*p*
*Citrulline*					
Basal	14.3 [11.4–19.4]	18.9 [15.4–21.8]	16.6 [12.1–33.4]	14.2 [10.7–20.8]	0.34
CGT	0.07 [0.05–0.11]	0.10 [0.07–0.13]	0.06 [0.03–0.11]	0.07 [0.05–0.11]	0.29

Numbers are N (%) and median [interquartile range]. CGT is expressed in µmol/L/min. CGT, citrulline generation test.

**Table 4:** Factors associated with four classes of citrulline generation test among 66 critically ill patients.

Variable	CGT <0.05 *n* = 21 (32)	CGT ≥0.05–<0.10 *n* = 23 (35)	CGT ≥0.10–<0.20 *n* = 16 (24)	CGT ≥0.20 *n* = 6 (9)	*p*
*Biological variables*					
Creatinine (µmol/L)	100 [65–120]	99 [77–144]	109 [72–149]	94 [51–133]	0.85
Creatinine clearance (mL/min)	63 [48–103]	60 [42–87]	62 [45–75]	69 [41–107]	0.84
C-reactive protein (mg/L)	134 [20–278]	73 [37–108]	59 [15–183]	35 [20–130]	0.43
Lactate (mmol/L)	2.0 [1.2–4.5]	1.6 [1.3–3.0]	1.9 [1.2–2.8]	1.7 [1.4–4.4]	0.88
*Amino acids*					
Glutamine 0 (µmol/L)	444 [379–554]	472 [370–640]	503 [355–682]	513 [462–591]	0.73
Glutamine 90 (µmol/L)	621 [540–818]	934 [734–1241]	1017 [787–1437]	1302 [1024–1367]	0.006
Citrulline 0 (µmol/L)	13.0 [9.5–20.3]	14.2 [11.1–17.3]	21.3 [15.6–29.7]	18.7 [14.5–21.9]	0.02
Citrulline 90 (µmol/L)	15.8 [12.0–23.5]	19.8 [17.6–24.3]	31.7 [27.5–41.7]	43.2 [41.0–43.3]	<0.001

Numbers are N (%) and median [interquartile range]. CGT is expressed in µmol/L/min. CGT, citrulline generation test.

**Table 5:** Correlation between the citrulline generation test and the biological variables of interest.

Variables of Interest	Correlation (R) with CGT	*p*
Age	0.23	0.06
C-reactive protein (mg/L)	−0.20	0.19
Creatinine (µmol/L)	0.07	0.58
Clearance of creatinine (mL/min)	−0.10	0.42
Basal citrulline (µmol/L)	0.28	0.02
Basal glutamine (µmol/L)	0.16	0.20
Peak glutamine (µmol/L)	0.46	<0.001

CGT, citrulline generation test.

**Table 6:** Factors associated with feeding intolerance among 66 critically ill patients.

Variable	Overall *n* = 66	Absence of FI *n* = 57	Presence of FI *n* = 9	*p*
*Clinical variables*				
Age (years)	67 [58–75]	66 [58–75]	68 [64–73]	0.41
Male sex	40 (61)	34 (60)	6 (67)	1
Heart rate (bpm)	86 [73–106]	86 [74–107]	85 [68–95]	0.48
Mean arterial pressure (mmHg)	84 [73–96]	87 [74–96]	77 [73–84]	0.27
*Indication for ICU admission*				
Cardiac arrest	*n* = 13	8 (14)	5 (56)	0.02
Neurological failure	*n* = 12	12 (21)	0 (0)	
Shock	*n* = 14	12 (21)	2 (22)	
Respiratory failure	*n* = 27	25 (44)	2 (22)	
*Biological variables*				
Creatinine (µmol/L)	101 [67–139]	98 [65–139]	111 [99–137]	0.25
Lactate (mmol/L)	1.7 [1.3–3.4]	1.6 [1.3–2.8]	4.5 [2.3–6.1]	0.03
*Variables of treatment*				
Norepinephrine use	23 (35)	19 (33)	4 (44)	0.71
Mechanical ventilation	47 (71)	39 (68)	8 (89)	0.43
*Variables of prognosis*				
SOFA score	8 [6–11]	8 [6–10]	13 [7–13]	0.15
28-day mortality	25/64 (39)	20 (36)	5 (56)	0.30
*Variables associated with the CGT*				
Glutamine (µmol/L)				
Baseline	481 [379–633]	472 [380–638]	490 [358–592]	0.62
Peak	877 [600–1264]	832 [600–1264]	1020 [748–1133]	0.72
Citrulline (µmol/L)				
Baseline	15.4 [11.9–21.6]	15.5 [12.7–21.6]	12.1 [10.8–21.3]	0.53
Peak	23.3 [16.2–33.7]	23.5 [17.7–33.7]	21.8 [14.4–30.6]	0.79
CGT	0.07 [0.04–0.12]	0.07 [0.04–0.11]	0.07 [0.06–0.17]	0.51

Numbers are N (%) and median [interquartile range]. CGT, citrulline generation test; FI, feeding intolerance, ICU, intensive care unit; SOFA, sequential organ failure assessment.

## Data Availability

The original contributions presented in this study are included in the article. Further inquiries can be directed to the corresponding author.

## Abbreviations

The following abbreviations are used in this manuscript:

AGI	Acute Gastro-Intestinal
CRP	C-Reactive Protein
CGT	Citrulline Generation Test
CVVHDF	Continuous Veno-Venous Hemodiafiltration
EN	Enteral Nutrition
FI	Feeding Intolerance
GRV	Gastric Residual Volume
ICU	Intensive Care Unit
SOFA	Sequential Organ Failure Assessment
